# Tea Polysaccharide Prevents Colitis-Associated Carcinogenesis in Mice by Inhibiting the Proliferation and Invasion of Tumor Cells

**DOI:** 10.3390/ijms19020506

**Published:** 2018-02-08

**Authors:** Li-Qiao Liu, Hai-Shan Li, Shao-Ping Nie, Ming-Yue Shen, Jie-Lun Hu, Ming-Yong Xie

**Affiliations:** 1 State Key Laboratory of Food Science and Technology, Nanchang University, Nanchang 330047, China; liuliqiao117@163.com (L.-Q.L.); lihaishan1112@163.com (H.-S.L.); shenmingyue1107@163.com (M.-Y.S.); hujielun2005@hotmail.com (J.-L.H.); 2 Basic Medical College, Nanchang University, Nanchang 330047, China

**Keywords:** tea polysaccharides, antitumor, colitis-associated cancer, cell proliferation, cell apoptosis

## Abstract

The imbalance between cell proliferation and apoptosis can lead to tumor progression, causing oncogenic transformation, abnormal cell proliferation and cell apoptosis suppression. Tea polysaccharide (TPS) is the major bioactive component in green tea, it has showed antioxidant, antitumor and anti-inflammatory bioactivities. In this study, the chemoprophylaxis effects of TPS on colitis-associated colon carcinogenesis, especially the cell apoptosis activation and inhibition effects on cell proliferation and invasion were analyzed. The azoxymethane/dextran sulfate sodium (AOM/DSS) was used to induce the colorectal carcinogenesis in mice. Results showed that the tumor incidence was reduced in TPS-treated AOM/DSS mice compared to AOM/DSS mice. TUNEL staining and Ki-67 immunohistochemistry staining showed that the TPS treatment increased significantly the cell apoptosis and decreased cell proliferation among AOM/DSS mice. Furthermore, TPS reduced the expression levels of the cell cycle protein cyclin D1, matrix metalloproteinase (MMP)-2, and MMP-9. In addition, in vitro studies showed that TPS, suppressed the proliferation and invasion of the mouse colon cancer cells. Overall, our findings demonstrated that TPS could be a potential agent in the treatment and/or prevention of colon tumor, which promoted the apoptosis and suppressed the proliferation and invasion of the mouse colon cancer cells via arresting cell cycle progression.

## 1. Introduction

The genetic mutations, chronic inflammation cases, epigenetic changes, poor diet and sedentary lifestyle are the key risk factors for colorectal cancer (CRC) [1]. Inflammation is one of the most common potential pathogens of carcinogenesis in the colon. The incidence of colitis-associated cancer (CAC) was closely associated with the ulcerative colitis [2]. Several epidemiological studies have shown a link between nutrition and certain kinds of cancers, including CRC [3]. Therefore, nontoxic phytochemicals are considered to be effective in tumor therapy [4].

China is known as the hometown of tea tree (*Camellia sinensis* L.O. Kuntze), which is a major tea producer globally. During the past two decades, it has been shown that *Camellia sinensis* L.O. Kuntze possesses various beneficial effects, including antioxidant, anti-inflammatory, anticancer, anticoagulation, anti-HIV and immune-enhancing activity. Many types of bioactive components have been found in tea [5,6]. Tea polysaccharides (TPS), as one of the major active ingredients of tea tree (*Camellia sinensis* L.O. Kuntze), have attracted a great deal of attention because of their various biological activities, such as antitumor, immunomodulation, antioxidant, anti-diabetes, radioprotection, and hepatoprotection [7,8,9,10]. Most of the coarse green tea leaves in low grade are thrown out for their awful taste and color, a large amount of the coarse leaves become unusable and overstocked after processing, which lead to not using these natural resources. While the coarse leaves are found to contain more TPS when compared with the tender tea leaves [11], the TPS content in older leaves is normally higher than that in young ones. Because TPS has diverse biological activities, it may be applied to the development of functional food. In most cases, TPS is found to be a glycoconjugate where protein carries one or more carbohydrate chains covalently attached to a polypeptide backbone, usually via *N*- or *O*-linkages [12]. Growing evidence suggests that TPS plays a vital function in human health. For example, TPS inhibits the carcinogenesis and tumor progression in mice and other tumor cell lines. In vitro, TPS showed anticancer activity against SKOV-3 cells [13]. Wei et al. reported that the polysaccharides from tea seeds suppressed the growth of K562 cells [14]. He et al. found the inhibitory effect of selenium-containing TPS on a breast cancer cell line, MCF-7 [15].

The study aimed to identify the possible antitumor effects of TPS using the AOM/DSS mouse model and mouse colorectal cancer cell line, CT26 cells. The molecular mechanism was further evaluated to test where TPS accelerated apoptosis and inhibited the proliferation of tumor cells.

## 2. Results

### 2.1. TPS Inhibited the Progression of Colitis Associated Colorectal (CAC) Cancer

**Figure 1:**
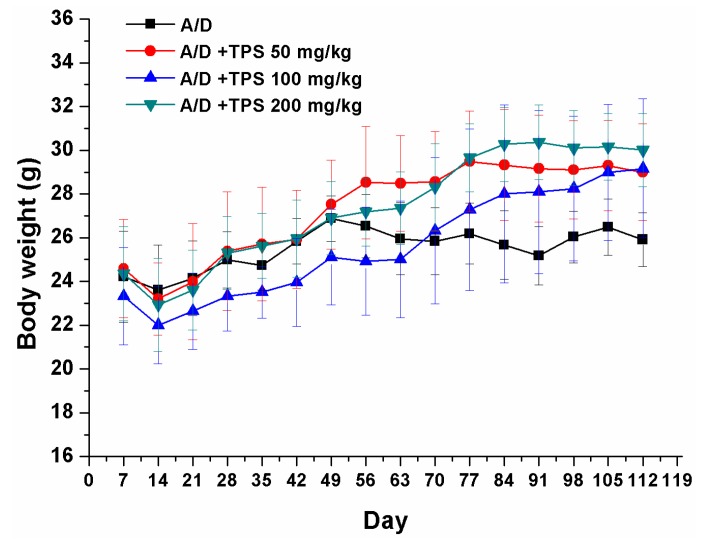
Base body weight changes of all groups after AOM/DSS (A/D) induction of CAC (*n* = 8 mice per group). The body weight loss in AOM/DSS group was observed throughout the study when the mice received 2% DSS in drinking water. However, TPS was found to increase the body weight in AOM/DSS-treated mice. Experimental Data in vivo were expressed as means ± SEM of at least three experiments.

The CAC mouse model was induced by an injection with procarcinogen AOM, followed by three cycles of DSS drinking water exposure to elicit colitis. As expected, the body weight loss in AOM/DSS group was observed throughout the study when the mice had 2% DSS in drinking water. In Figure 1, TPS plays an important role in regaining body weight in AOM/DSS-treated mice. After the mice were killed, multiple colonic tumors were found in all the mice that received AOM/DSS, and the tumors were confined to the middle and distal colon. The number of tumors in the AOM/DSS mice was more than those in TPS-treated AOM/DSS mice (Table 1). The treatment with 200 mg/kg TPS resulted in a reduction of the tumor incidence in AOM/DSS-treated mice (*p* < 0.05), suggesting that TPS showed outstanding antitumor activity in the AOM/DSS-treated mice. Besides, the colons of the TPS-treated AOM/DSS group mice were relatively longer than those of the AOM/DSS group mice (Table 1). Also, it was found that TPS was well tolerated in mice and there was no observable toxicity and gross changes in all groups up to 112 days (Table 2). TPS at different doses showed a significant impact on the spleen and thymus indexes. In this study, it was observed that AOM/DSS treatment significantly increased the mice’s spleen weight. The increase in the spleen index may be brought by the inflammation state of AOM/DSS-treated mice where the function of the immune cells and the inflammatory responses were activated.

### 2.2. TPS-Induced Apoptosis and Inhibited Cell Cycle Progression in CAC Mice

**Figure 2:**
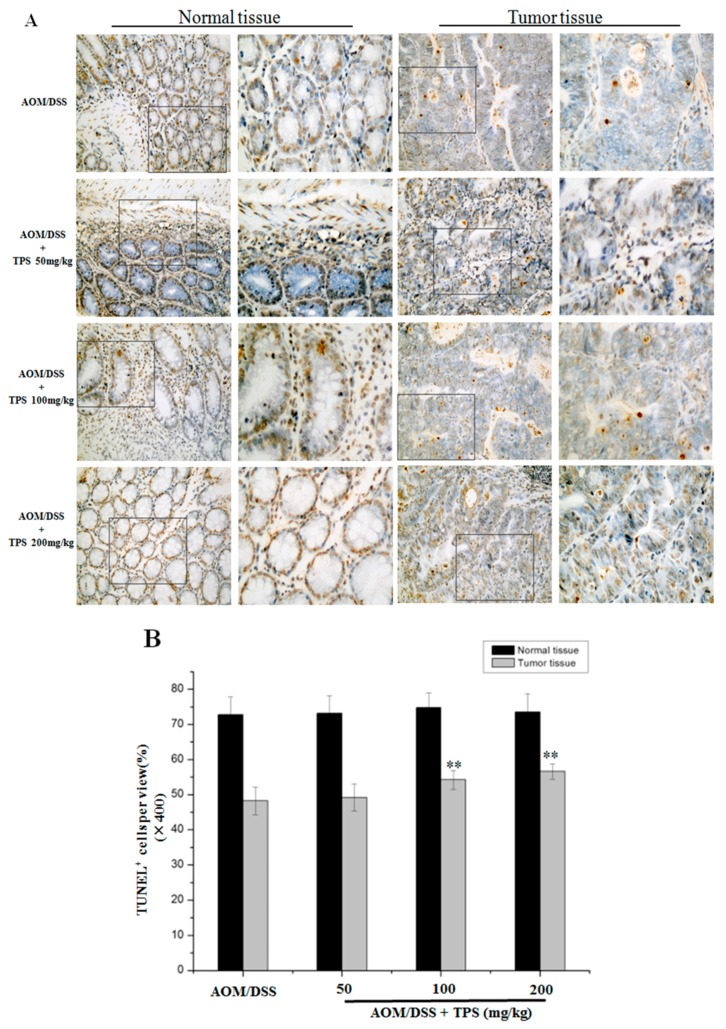
Paraffin-embedded colon sections were stained with a TUNEL assay for assessment of apoptosis. (**A**) The increased number of apoptotic cells was observed in the tumor tissues of TPS-treated AOM/DSS mice compared with the AOM/DSS mice. Representative TUNEL stained images in tumor tissues and normal tissues (*n* = 6) (original magnification 200× and 400×). (**B**) The number of TUNEL-positive cells per field (400×, *n* = 10) was significantly increased in colonic tumor tissues of TPS-treated AOM/DSS mice compared with the AOM/DSS mice. Bar graphs show mean ± SD of three independent experiments. ** *p* < 0.01, vs. AOM/DSS group.

**Figure 3:**
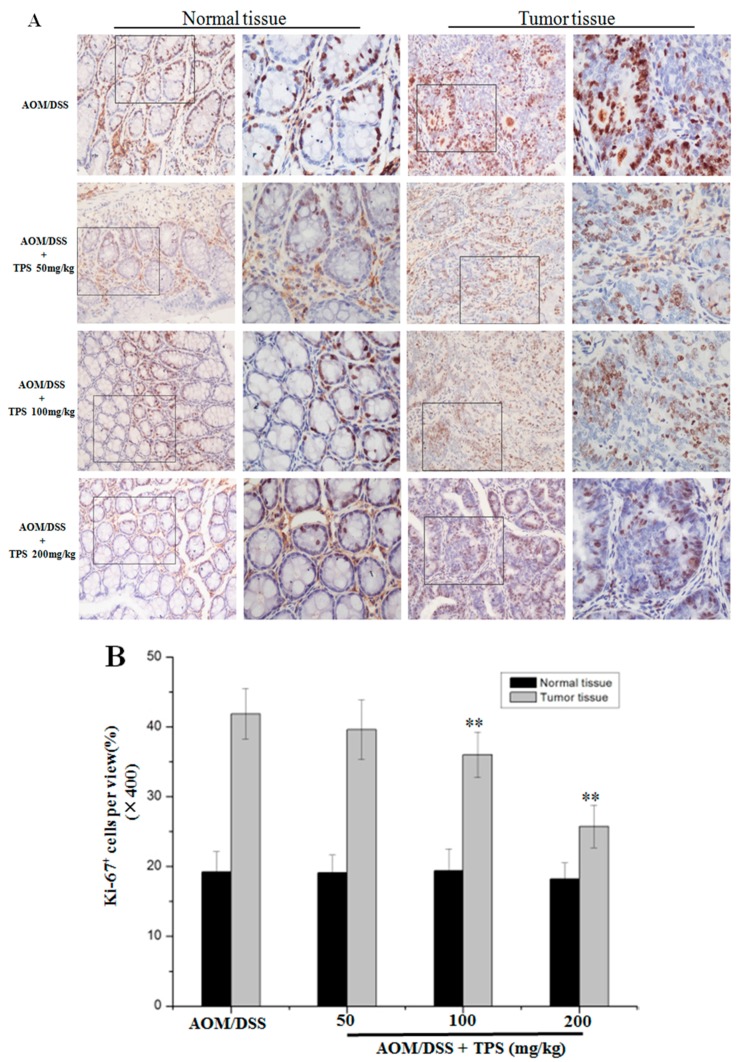
Paraffin-embedded colon sections were stained with Ki-67 for assessment of proliferation. (**A**) TPS significantly altered expression of Ki-67. Representative images from immunostaining of tumor tissue samples (*n* = 6) with antibodies against Ki-67 (original magnification 200× and 400×). (**B**) Quantification of Ki-67-positive cells per view (400×, *n* = 10) showed that the number of Ki-67 + cells was significantly decreased in colonic tumor tissues of TPS-treated mice compared with AOM/DSS mice. Bar graphs show mean ± SD of three independent experiments. ** *p* < 0.01, vs. AOM/DSS group.

The effect of TPS treatment was analyzed on cancer cell apoptosis and cell proliferation in the AOM/DSS mice. TdT-mediated dUTP-biotin nick end labeling (TUNEL) staining in colonic crypt cells and tumor epithelia showed that TPS increased apoptosis compared with the AOM/DSS group (Figure 2). The treatment with 200 mg/kg TPS in the AOM/DSS mice led to an 8.38% increase of apoptosis in tumor tissue areas compared with the non-treated AOM/DSS mice. Also, no significant difference in the apoptosis of crypt cells in normal tissue was observed (Figure 2). In assessing the effect of TPS on cell proliferation, Ki-67 expression was identified in the colonic crypt cells and tumor epithelia by immunohistochemical staining. In relation to the results of tumor numbers, the TPS treatment led to a substantial reduction of Ki-67-labeled cells in AOM/DSS mice (Figure 3). The treatment with 200 mg/kg TPS in AOM/DSS mice led to a 16.13% decrease in tumor tissue areas. However, no significant difference in proliferation of crypt cells in the normal tissue was observed (Figure 3). In summary, the data indicated that TPS inhibited AOM/DSS-induced development of the CAC cancer.

### 2.3. TPS Inhibited the Expression of Cyclin D1, MMP-2, and MMP-9 in CAC Mice

**Figure 4:**
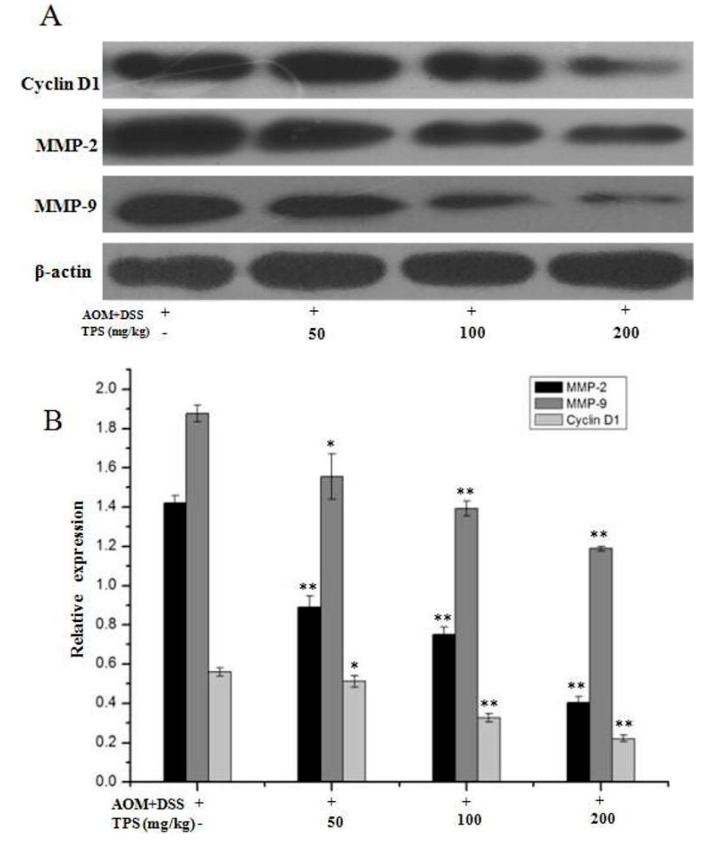
Protein expression levels of cyclin D1, MMP-2, and MMP-9 in AOM/DSS mice by Western blot. (**A**) Western blots were performed to see the effects of TPS on the expression of proteins cyclin D1, MMP-2, and MMP-9. (**B**) Densitometric analysis was performed to determine the relative levels of the proteins cyclin D1, MMP-2, and MMP-9. The results are expressed as mean ± SD of three independent experiments. * *p* < 0.05, ** *p* < 0.01, vs. AOM/DSS mice.

The aforementioned results showed that TPS led to a decrease in cellular proliferation and cell invasion and induced cell apoptosis. As an essential role of the cell cycle and invasion in the CAC development, the protein expression levels were further evaluated in these CAC mice. In Figure 4, TPS significantly inhibited the expression of cell cycle protein cyclin D1, a cell cycle protein necessary for the G_1_/S phase progression, invasion-related proteins, MMP-2 and MMP-9.

### 2.4. Effect of TPS on Cell Proliferation and Invasion of CT26 Cells In Vitro

**Figure 5:**
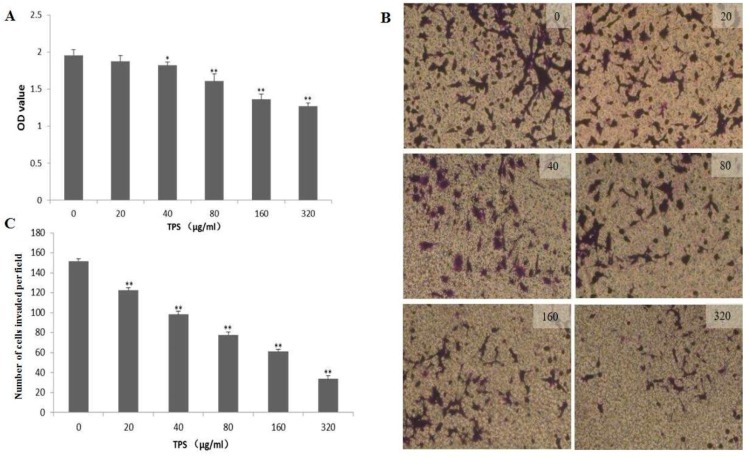
(**A**) Effect of TPS on the proliferation of CT26 cells. CT26 cells were seeded into 96-well culture plates and treated with TPS at different concentrations for 48 h. The proliferation of CT26 cells was evaluated using CCK8 assays. Bar graphs show mean ± SD of three independent experiments. * *p* < 0.05, ** *p* < 0.01 vs. control group. (**B**) TPS inhibited the invasion ability of CT26 cells. The CT26 cells were seeded into 24-well Matrigel-coated transwell chambers and incubated at 37 °C for 48 h with TPS at different concentrations. (**C**) Bar graphs show the results of Boyden chamber assays, TPS significantly inhibited the CT26 cells’ invasion in a dose-dependent manner. The results are expressed as mean ± SD of three independent experiments. * *p* < 0.05, ** *p* < 0.01 vs. control group.

**Figure 6:**
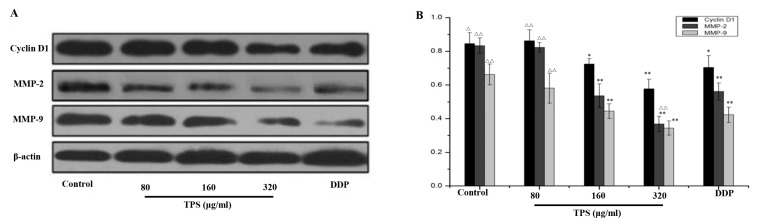
(**A**) Expression of the proteins cyclin D1, MMP-2, and MMP-9 in CT26 cells by Western blots. (**B**) Densitometric analysis was performed to determine the relative levels of cyclin D1, MMP-2, and MMP-9. Bar graphs show mean ± SD of three independent experiments. * *p* < 0.05, ** *p* < 0.01 vs. control group. ^△^ *p* < 0.05, ^△△^ *p* < 0.01 vs. DDP group.

To determine whether TPS inhibited the proliferation and invasion of CT26 cells, CCK-8 assay and Boyden chamber assays were used to evaluate the cellular viability and invasiveness. The CT26 cells were inoculated into a 96-well plate and treated with different concentrations of TPS for 48 h. In Figure 5A, TPS at concentrations of 20–320 μg/mL significantly decreased the proliferation of the CT26 cells compared with the control group. Boyden chamber assays showed that the invasion of the CT26 cells was decreased by treating with TPS at concentrations of 20–320 μg/mL for 48 h (Figure 5B). The results of the CCK-8 assay and Boyden chamber assays showed that TPS significantly inhibited the CT26 cells’ proliferation and invasion in a dose-dependent manner (Figure 5C). As seen in Figure 6, TPS significantly depressed the level of cyclin D1 and invasion-related proteins MMP-2 and MMP-9. These results suggested that TPS could inhibit the CT26 cells’ invasion. 

### 2.5. TPS-Induced Apoptosis and Inhibited Cell Cycle Progression of CT26 Cells In Vitro

**Figure 7:**
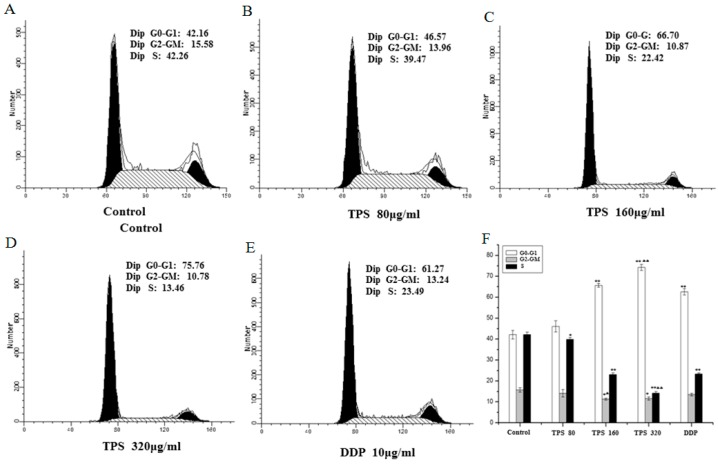
TPS provoked apoptosis in CT26 cells. Apoptosis rates of the control group, TPS group at indicated concentrations and positive control group DDP cells for 48 h were determined by FACS analysis (**A**–**E**). (**F**) Bar graphs show mean ± SD of three independent experiments. ** *p* < 0.01, vs. control group. ^ΔΔ^ *p* < 0.01 vs. DDP group.

**Figure 8:**
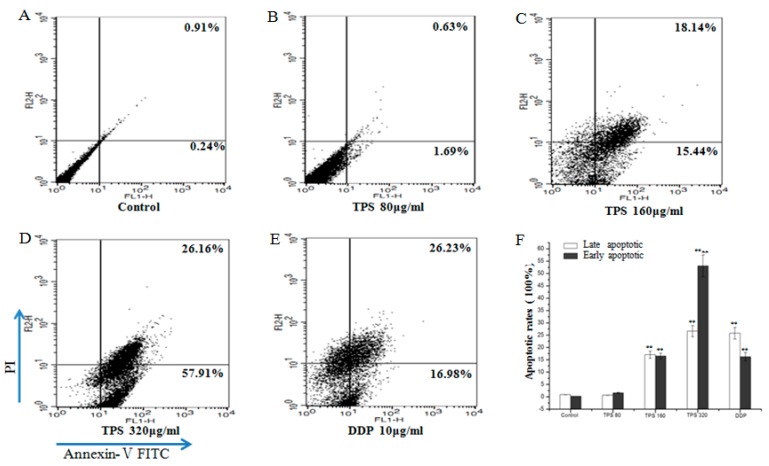
TPS inhibited cell cycle in CT26 cells. Cell cycle progression of the control group, TPS group at indicated concentrations and positive control group DDP cells for 48 h were determined by FACS analysis (**A**–**E**). (**F**) Bar graphs show mean ± SD of three independent experiments. * p < 0.05, ** p < 0.01, vs. control group. ^Δ^ *p* < 0.01, ^ΔΔ^ *p* < 0.01 vs. DDP group.

The results showed a significant effect of TPS on cell proliferation at 80–320 μg/mL. The role of TPS was further investigated in cell cycle progression using flow cytometry. The disorder of the cell cycle regulation was frequently related to the abnormal proliferation and differentiation of tumor cells [16,17]. Hence, it was assessed whether TPS may induce cell cycle arrest and finally led to apoptosis. The CT26 cells were treated with TPS at 80 μg/mL, 160 μg/mL and 320 μg/mL for 48 h and then used for cell cycle analysis. The apparent cell cycle arrest in G_0_/G_1_ phase at 80–320 μg/mL TPS-treated cells were observed when compared with the control group (Figure 7). The apoptosis rates of early stages ranged from 1.69% to 57.91% and maximum effect was found in TPS group at a dose of 320 μg/mL, suggesting that the intervention of TPS induced apoptosis (Figure 8). The results showed that TPS-induced apoptosis and the status may be mediated through cell cycle blockage.

## 3. Discussion

Numerous health beneficial effects with considerable potential have been attributed to tea, however, probably the most remarkable and controversial remains that of anti-tumor activity. In recent years, the health benefits of green tea including anticancer activity have been widely reported [18,19,20,21]. The tea leaves have abundant chemical components such as polyphenols, proteins, and polysaccharides (TPS). Reports on the TPS extracted from tea leaves have been on an upward trend [22,23]. The polysaccharides are widely found in nature, especially in higher plants and edible fungi and possess a wide range of bioactivities with relatively low toxicity, attracting so much attention in the field of life science [24,25]. For instance, *Lentinan* showed an effective antitumor activity and significantly increased chemotherapy sensitivity, and played an important role in improving the immune function and life quality of patients when combined with chemotherapy [26]. Previous studies have found that polysaccharides from *Ganoderma atrum* (PSG-1) contained potential antitumor activity and activated host immune responses in vitro and in vivo [27,28].

In this study, it was demonstrated that TPS inhibited colon cancer cell proliferation, tumorigenesis and CAC progression in mice with no side effects, through both in vivo and in vitro assays. The study focused on evaluating the underlying molecular mechanism where TPS repressed tumor growth in CAC mice and proliferation of CT26 cells. To exhibit the function of TPS in vivo, the well-established AOM/DSS mouse model was employed [29]. It is widely used in the analysis of colon cancer. Oral administration of TPS significantly suppressed the tumor progression in CAC mice (Table 1), showing that TPS has potential anticancer activity. It was then speculated that the therapeutic intervention of TPS might trigger the relevant molecular mechanisms to inhibit tumorigenesis. The unlimited progression is considered a consequence of many biochemical processes in the tumor cell, such as the abnormal regulation of gene expression. The apoptosis and invasion have reportedly contributed to a high cell mutation rate in malignant derivatives [30]. There was an imbalance between cell apoptosis and proliferation that resulted in tumor progression. An uncontrolled cell proliferation caused cell cycle deregulation, relating to G_1_/S checkpoint markers that were in charge of cell cycle progression. The TUNEL assay showed that TPS increased the apoptosis ratio in AOM/DSS mice in a concentration-dependent manner (Figure 2). The Ki-67 expression in tumor epithelia tends to be reduced in TPS-treated CAC mice compared with AOM/DSS group (Figure 3). These results may be induced by TPS-induced apoptosis, and the antitumor effect of TPS could be mediated by cell cycle suppression.

It is reported that the overexpressed cyclin D1 has association with an increase not only in tumor progression but also in poor outcome of patients, and downregulation of cyclin D1 expression may be a potential treatment for malignant neoplasms [31,32]. The cyclin D1 mainly regulates the phase G_0_–G_1_, and activates the G_1_/S transition. In addition, it has been widely recognized as a kind of proto-oncogenes and its excessive expression can cause cell proliferation and malignant change. It has been demonstrated that the increased cyclin D1 expression was associated with the malignant transformation in different tumor cell lines, such as lung, liver and prostate [33,34,35,36]. In this study, the level of cyclin D1 protein was found decreased gradually with the oral administration of TPS at 50–200 mg/kg. The results indicated that TPS could inhibit cell proliferation by arresting the cell cycle through regulating cyclin D1 expression (Figure 4).

The migration was brought by the growth of cancer cells while the tumor metastasis involves a series of the sequential steps, including cell proliferation, migration, invasion, adhesion and vessel formation [37]. It was found that TPS downregulated the expression of metastasis genes (Figure 6). Both MMP-2 and MMP-9 were reported to degrade the basement membrane (BM), playing a key role in cancer cell invasion and metastasis [38]. Therefore, the inhibition of MMPs, particularly MMP-2 and MMP-9, is being used for anticancer therapy. In this research, the expression levels of MMP-2 and MMP-9 were reduced by TPS. These results showed the possibility that TPS may inhibit tumorigenesis by downregulating the levels of the cycle protein cyclin D1, MMP-2 and MMP-9, which are necessary for tumor development.

Many studies noted the immunoregulation activity of polysaccharides [39,40,41,42]. The organ weights like the thymus and spleen, where their index reflects the immune function and immune status of the body and the assessment of immunotoxicity. The results confirmed that TPS may ameliorate nonspecific immunity even when the body suffered from tumors. Also, it was found that AOM/DSS treatment induced inflammatory responses among mice. However, the treatment with 200 mg/kg TPS showed an outstanding anti-inflammatory activity in AOM/DSS-treated mice. In addition, clinical studies noted that the most effective anticancer treatment was related to the triggers of an effective antitumor immune response [43].

Further work was focused on identifying the effect of TPS on CT26 cells and clarifying the underlying molecular mechanism. TPS treatment significantly reduced the cell viability and induced apoptosis in CT26 cells in a concentration-dependent approach. To provide insights into the mechanism by which TPS inhibited colon cancer, the expression of the cell cycle progression related proteins was analyzed and found that TPS dramatically reduced the S-phase percentage and depressed cyclin D1 expression. These results suggested that TPS could regulate cell proliferation through the modulation of the cyclin D1 expression. Also, it was found that TPS decreased the invading ability of the colorectal cancer cell line CT26, and the MMP-2 and MMP-9 protein expression levels were reduced by TPS treatment. Both MMP-2 and MMP-9 are vital mediators of the invasion in many cancer types. Hence, restraining MMP-2 and MMP-9 levels could influence the invasion and metastasis of the tumor cells, which may lead to the tumor cell apoptosis and interruption of the colon carcinogenic process.

In conclusion, the research has found that TPS restrained cell proliferation in CT26 cells and CAC mice. The underlying mechanism could involve promoting apoptosis, inhibiting invasion and cell proliferation of tumor cells and promoting the host immune response. TPS exhibited its biological activity by direct antitumor effects, such as the induction of apoptosis, cell cycle arrest and invasion of the murine tumor cell line CT26. Therefore, TPS could be one of the phytochemicals that may be used in treating the colon tumors. Many kinds of polysaccharides are used in the auxiliary treatment of the tumors that have shown a good application potential. However, the structure of TPS is complex and its antitumor mechanism of polysaccharides is unclear. Therefore, further studies of TPS structure and the structure-activity relationship may help to clarify the antitumor mechanism of TPS and its significance in tumor therapy in the future.

## 4. Materials and Methods

### 4.1. Reagents

TPS was extracted and purified from the collected coarse tea leaves harvested from Wuyuan, Jiangxi Province, China. The tea leaves were soaked in 80% alcohol with the ratio of 1:10 g/mL for 24 h, residue was dried and digested in hot water at 95 °C for 4 h. After being extracted twice, alcohol was used to precipitate the tea coarse polysaccharides. The extract was then deproteinized using the Sevag reagent, then by a chloroform-*n*-butyl alcohol mixture at a ratio of 4:1 (*v*/*v*). The deproteinization process was carried out three times. The extract was then dialyzed against distilled water for 72 h, concentrated by vacuum and then freeze-dried [44]. It was homogeneous polysaccharides by the HPGPC (High Performance Permeation Chromatography) analysis. The relative molecular mass was 289,734 Da. The GC (Gas Chromatography) analysis confirmed that tea polysaccharides were mainly composed of rhamnose, ribose, arabinose, mannose, glucose and galactose. Their molar ratio is 1.26:3.18:4.08:1.00:1.52:3.29.

Azoxymethane (AOM) was bought from Sigma-Aldrich (St. Louis, MO, USA). The low-molecular weight dextran sulfate sodium (DSS) was from MP Biomedicals Inc. (Irvine, CA, USA). Positive control group drug Cisplatin (DDP) was taken from Qilu Pharmaceutical Co.; Ltd. (Shandong, China).

### 4.2. Cell Line and Conditioned Culture

The murine tumor cell line CT26 was from the Cell Bank of Type Culture Collection of Chinese Academy of Sciences, Shanghai, China. The CT26 cells were cultured in a medium which was made up of RPMI-1640 medium (Hycolne, Logan, UT, USA), 10% fetal bovine serum (FBS) (Hycolne, Logan, UT, USA), 100 units/mL penicillin and 100 units/mL streptomycin. The cells were incubated in a humidified atmosphere with 5% CO_2_ at 37 °C.

### 4.3. Cell Viability and Proliferation Assays

The effect on survival ability of the CT26 cells was counted using the Cell Counting Kit-8 (CCK-8, Dojindo Laboratories, Kumamoto, Japan), the cells were inoculated into a 96-well plate (1.0 × 10^4^ cells/mL) containing different amounts of TPS (20 μg/mL, 40 μg/mL, 80 μg/mL, 160 μg/mL and 320 μg/mL). CT26 cells of the control group were cultured in RPMI-1640 medium without TPS. After incubation for 48 h, 10 μL of CCK-8 reagent was added, cultivating the CT26 cells at 37 °C for 1 h. The absorbance was measured by using a microplate reader Thermo Scientific Varioskan Flash (Thermo Fisher Scientific, Waltham, MA, USA) at 450 nm.

### 4.4. Assessment of Cell Apoptosis by Annexin V-FITC/PI Double-Staining Assay

The CT26 cells were stained with Annexin V-FITC/PI detection kit (Vazyme, Nanjing, China) using the manufacturer’s instructions for the identification of apoptosis rate. Then, the cells were harvested, resuspended and mixed with a buffer containing the Annexin V-FITC/PI fluorescence dye for 15 min without light at 37 °C. The stained CT26 cells were washed completely with PBS, flow cytometry (FACSCalibur, Becton Dickinson, Franklin Lakes, NJ, USA) was used to analyze the cell apoptosis.

### 4.5. Analysis of Cell Cycle

To evaluate the cell cycle of the CT26 cells, the cells were stained with propidium iodide (Vazyme, Nanjing, China) followed the manufacturer’s instructions. Then, the CT26 cells were harvested, resuspended and mixed with a buffer containing the propidium iodide fluorescence dye for 30 min without light at 37 °C. The cell cycle was analyzed by flow cytometry (FACSCalibur, Becton Dickinson, Franklin Lakes, NJ, USA).

### 4.6. Cell Matrigel Invasion Assay

The CT26 cells’ invasiveness was analyzed using the Boyden chamber assays. Then, the upper surface of the filter (8.0 μm pore size) was coated with 40 μg of 0.5% Matrigel (BD Biosciences, San Jose, CA, USA) overnight at room temperature. In the upper chamber, about 1 × 10^4^ CT26 cells were cultivated with a serum-free RPMI-1640 medium, while RPMI-1640 medium containing 10% FBS was added to the lower chamber. Different amounts of TPS (20 μg/mL, 40 μg/mL, 80 μg/mL, 160 μg/mL and 320 μg/mL) were added into the upper chamber. After 48 h, the filters were fixed and the invaded cells were stained using crystal violet. The number of invading CT26 cells was counted with under a light microscope.

### 4.7. AOM/DSS-Induced Colitis-Associated Colorectal Carcinogenesis and TPS Treatment

BALB/c mice (20.0 ± 2.0 g) were provided by Medical Laboratory Animal Center, Nanchang University. The research animals were maintained based on the guidelines published in the Guide for the Care and Use of Laboratory Animals (NRC 2011). The animal experiment was approved by the First Affiliated Hospital Ethnics Committee of Nanchang University (No. 20140312, 12 March 2014). The mice were kept in plastic cages with woodchip bedding, and were fed by standard diet and water ad libitum. They were kept at a constant 25 °C under a 12 h light/12 h dark cycle.

The AOM/DSS mouse model is widely used in the analysis of molecular mechanism involved in the inflammation-correlated cancerization [45]. The animals were intraperitoneally injected with AOM [10 mg/kg initial body weight (BW)] and maintained at a standard diet and water for 7 d. After which, 2% DSS was added to the drinking water for the next 7 d. Then the mice were given a normal drinking water ad libitum for 14 d and subjected to two more 2% DSS treatment cycles. The AOM/DSS mice were then classified randomly into four groups (*n* = 10). The animals received TPS treatment every day via gastric tubes at doses of 50 mg/kg, 100 mg/kg and 200 mg/kg BW from Week 4 until the end of the animal experiment. The negative control group and AOM/DSS group were treated with 0.9% saline. The BWs were monitored three times per week throughout the experiment. On Day 112, the mice were killed and their colon tissues, spleen, and thymus were taken. Their spleen and thymus indexes were presented as the spleen and thymus weight relative to BW. The colons in mice were opened longitudinally and washed with phosphate-buffered saline (PBS), and megascopic tumors were calculated and surveyed with a caliper.

### 4.8. Immunohistochemistry

The Ki67 expression in colon paraffin sections was determined by its immunohistochemistry. The paraffin-embedded thin-tissue sections with a 5μm thickness were rehydrated initially in a xylene and then in graded ethanol solutions. The sections were then mixed with HistoVT (10×, pH 7.0) antigen repair solution for 20 min at 90 °C and cooled down to room temperature. The sections were blocked with 5% bovine serum albumin (BSA) in TBST for 2 h, and the slides were then incubated with primary antibodies, Ki-67 rabbit polyclonal antibody (ab15580, 1:200 dilution, Abcam, Cambridge, UK) overnight at 4 °C. After the sections were washed for three times with TBST, the slides were co-incubated with matched secondary antibodies (ZB2301, 1:2000 dilution, ZSGB-BIO, Beijing, China) diluted with 5% BSA in TBST for 2 h at room temperature. Then the slides were washed with TBST and incubated for 10 min using DAB kit in accordance with the manufacturer’s instructions (Zsbio, Beijing, China). The counter-staining was carried out using the hematoxylin (Zsbio, Beijing, China), and the sections were observed using the light microscope (Olympus, Tokyo, Japan).

### 4.9. Terminal Deoxynucleotide Transferase (TdT) dUTP Nick End Labeling (TUNEL) Assay

The TUNEL method was used in the paraffin-embedded thin tissue sections (5 μm thick), where the TUNEL apoptosis detection kit (Roche, Indianapolis, IN, USA) was used to identify the cell apoptosis status following the manufacturer’s instructions. The images were captured with a light microscope (Olympus, Tokyo, Japan).

### 4.10. Western Blot Analysis

The extracted protein from 10% tissue homogenate was used to evaluate the expression level of cell cycle regulator, cyclin D1 and invasiveness-related proteins, MMP-2 and MMP-9. The total protein from colon tissues was separated by SDS-PAGE (Sodium dodecyl sulfate polyacrylamide gel electrophoresis) electrophoresis and electrotransferred to a PVDF (Polyvinylidene Fluoride) membrane. The PVDF membranes were blocked using TBST, which contains 5% BSA for 2.5 h at room temperature. They were further incubated for 2 h with anti-cyclin D1 rabbit monoclonal antibody (#2978, 1:1000 dilution, Cell Signaling Technology, Tokyo, Japan), anti-MMP-2 goat polyclonal antibody (AF1488, 1:1000 dilution, R&D Systems, Minneapolis, MN, USA), anti-MMP-9 goat polyclonal antibody (AF909, 1:1000 dilution, R D Systems, Minneapolis, MN, USA) and anti-β-actin mouse monoclonal antibody (TA09, 1:2000 dilution, ZSGB-BIO, Beijing, China). After washing three times with TBST, the PVDF membranes were incubated with an HRP-conjugated secondary antibody (ZB-2306, ZB2301, 1:2000 dilution, ZSGB-BIO, Beijing, China) for 1.5 h at room temperature. The PVDF membrane was washed three times with TBST, and the immunoreactive protein was visualized by using the enhanced chemiluminescent reagent (Supersignal Chemiluminescent Substrate, Pierce, Rockford, IL, USA).

### 4.11. Statistical Analysis

Experimental Data in vitro were expressed as the means ± SD and in vivo were expressed as means ± SEM of at least three experiments. One-way ANOVA was used to evaluate the difference between three or more groups of the study for multiple comparisons. All statistical analysis was performed by SPSS 17.0 software (SPSS, Inc., Chicago, IL, USA). The differences were deemed to be statistically distinct at *p* values < 0.05.

## Figures and Tables

**Table 1:** Colon length and tumor number at the end of the experiment.

Group	Dose (mg/kg)	Colon Length (cm)	Tumor Number
AOM/DSS	0	6.582 ± 0.250	16.667 ± 2.582
	50	7.117 ± 0.380	11.667 ± 2.160 ^Δ^
AOM/DSS + TPS	100	7.747 ± 0.318 ^ΔΔ^	8.833 ± 1.472 ^ΔΔ^
	200	8.272 ± 0.450 ^ΔΔ^	4.833 ± 1.722 ^ΔΔ^

Each data point represents the mean ± SEM of at least three experiments (*n* = 8 mice per group). ^Δ^ *p* < 0.05, ^ΔΔ^ *p* < 0.01 vs. AOM/DSS mice.

**Table 2:** Effect of TPS on spleen index and thymus index in AOM/DSS mice and normal mice.

Group	Dose (mg/kg)	Spleen Index (mg/g)	Thymus Index (mg/g)
Normal	200	5.732 ± 0.359 ^ΔΔ^	1.773 ± 0.051 ^ΔΔ^
Normal + TPS	0	6.455 ± 0.341 **^Δ^	2.232 ± 0.090 **^ΔΔ^
AOM/DSS	0	7.035 ± 0.206 **	1.957 ± 0.181
	50	6.843 ± 0.262 **	2.402 ± 0.250 **^ΔΔ^
AOM/DSS + TPS	100	6.672 ± 0.229 **	2.408 ± 0.116 **^ΔΔ^
	200	6.510 ± 0.256 *^Δ^	2.672 ± 0.093 **^ΔΔ^

Each data point represents the mean ± SD of six animals for each group. * *p* < 0.05, ** *p* < 0.01, vs. Normal mice. ^Δ^ *p* < 0.05, ^ΔΔ^ *p* < 0.01 vs. AOM/DSS mice.
